# Association of CT and MRI Manifestations with Pathology in Dysembryoplastic Neuroepithelial Tumors

**DOI:** 10.5334/jbsr.2940

**Published:** 2022-12-23

**Authors:** Da-wei Liao, Xue Zheng, Qian-qian Feng, Tian Xia

**Affiliations:** 1 The Affiliated Hospital of Southwest Medical University, CN

**Keywords:** Dysembryoplastic neuroepithelial tumor, CT, MRI, Pathology

## Abstract

**Objective:**

To investigate the CT, MRI and pathological features of dysembryoplastic neuroepithelial tumor (DNET).

**Methods:**

The CT and MRI features of six cases of pathologically confirmed DNET were retrospectively analyzed and compared with pathology.

**Results:**

All six cases of DNET were solitary, and lesion in one case was located in the right parietal lobe, one case in the right frontal lobe, one case in the cerebellar vermis, one case in the right temporo-parietal occipital lobe, one case in the left basal ganglia, and one case in the pineal gland. CT and MRI showed cystic solid tumor in all six cases, of which four showed calcification. In CT images, the cystic components appeared low-density, solid nodules, septa, and cyst walls were slightly high-density, and calcifications were high-density. In MRI images, the cystic components were hypointense on T1WI and hyperintense on T2WI, solid nodules, septa and cyst walls were iso-intense or slightly hyperintense on T1WI and T2WI sequences, and calcifications were all hypointense. On enhanced scan, the cystic components were not enhanced, and the solid nodules, septa, and cyst walls were inhomogeneously enhanced.

**Conclusion:**

The imaging manifestations of DNET are characteristic, and the combination of clinical and imaging features can greatly improve diagnostic accuracy.

## Introduction

Dysembryoplastic neuroepithelial tumor (DNET) is a rare benign tumor of the central nervous system, accounting for 0.2%–1.2% of neuroepithelial tumors [1]. In 1988, Daumas-Duport et al. [2] first proposed and comprehensively analyzed DNET, which causes early onset of epilepsy in children and young adults. In the 2016 edition of the World Health Organization (WHO) classification of tumors of the central nervous system, DNETs are classified as grade I neuronal-mixed neuronal tumors according to the WHO classification [3]. We retrospectively analyzed the clinical, pathological, CT and MRI manifestations of six patients with DNET confirmed by surgery and pathology in our hospital, aiming to improve the diagnostic accuracy based on DNET imaging.

## 1 Materials and methods

### 1.1 General information

Six patients with DNET were confirmed by surgery and pathology in our hospital from December 2017 to December 2021. Four males and two females were aged 8–29 years, with an average age of 14.2 years. Five patients had epilepsy as their first symptom and were poorly treated with antiepileptic drugs. According to the classification criteria of the International League Against Epilepsy [4], there were three cases of generalized seizures and two cases of partial seizures. One patient had dizziness, headache, and vomiting for two days. There was no recurrence in the follow-up of post-surgery patients. All subjects separately agreed in writing to participate in the study.

### 1.2 Examination methods

All six patients underwent MRI, CT plain scan and enhanced scan. MRI examinations were performed in a 3.0 Tesla magnet (Achieva, PHILIPS), and 8-channel head coil was selected. Transverse axial T1WI, T2WI, FLAIR, sagittal T1WI and diffusion weighted imaging (DWI) were routinely scanned. T1WI and T2WI were TSE sequences, T1WI repetition time (TR), echo time (TE) were 488 ms and 15 ms, respectively; T2WI TR and TE were 3751ms and 100 ms, respectively; T2WI-FLAIR TR and TE were 7500 ms and 100 ms, respectively; DWI TR and TE were 2678.59 ms and 98.84 ms, respectively. The dispersion sensitivity coefficients (b-value) were 0 s/mm^2^ and 1000 s/mm^2^, respectively; The acquisition matrix was 512 × 512, the FOV was 23 × 23 cm, the layer thickness was 5.5 mm, the layer spacing was 1 mm, and the deflection angle (FA) was 90°. At the same time, Gadobenate Dimeglumine Injection (0.1 mm per kg body weight) was injected into the cubital vein for enhanced scanning at a rate of 2.0 mml/s. T1WI-FFE sequence and TR/TE (155.60/2.44 ms) were used for transverse, coronal, and sagittal scanning.

CT scans were performed using a PHILIPS 256-slice Brilliance iCT machine. Voltage: 120 KV, tube current: 240 mAs, scanning matrix: 512 × 512, layer thickness at 5 mm, pitch ratio of 0.392, FOV = 25 × 25 cm. Enhanced scanning was performed using a high-pressure syringe. Contrast agent Iohexel Injection (300 mgI/mL) 75–80 mml was bolus injected at a rate of 5–6 ml/s. Continuous upward scanning was performed with the auditory canthal line (OM line) as the baseline at supine position.

### 1.3 Image Analysis

All images were read by two chief physicians with experienced in the head and neck imaging using a single-blind method. The two chief physicians have been working for 20 years, and each year, there are 1,000 head and neck MRI readings. The location, shape, edge, adjacent relationship, signal or density, and enhancement characteristics of the lesions were analyzed in detail. Consensus was reached after discussion if there was a disagreement. The common signs of the two chief physicians were used as reliable signs.

## 2 Results

### 2.1 Morphological features

**Figure 1:**
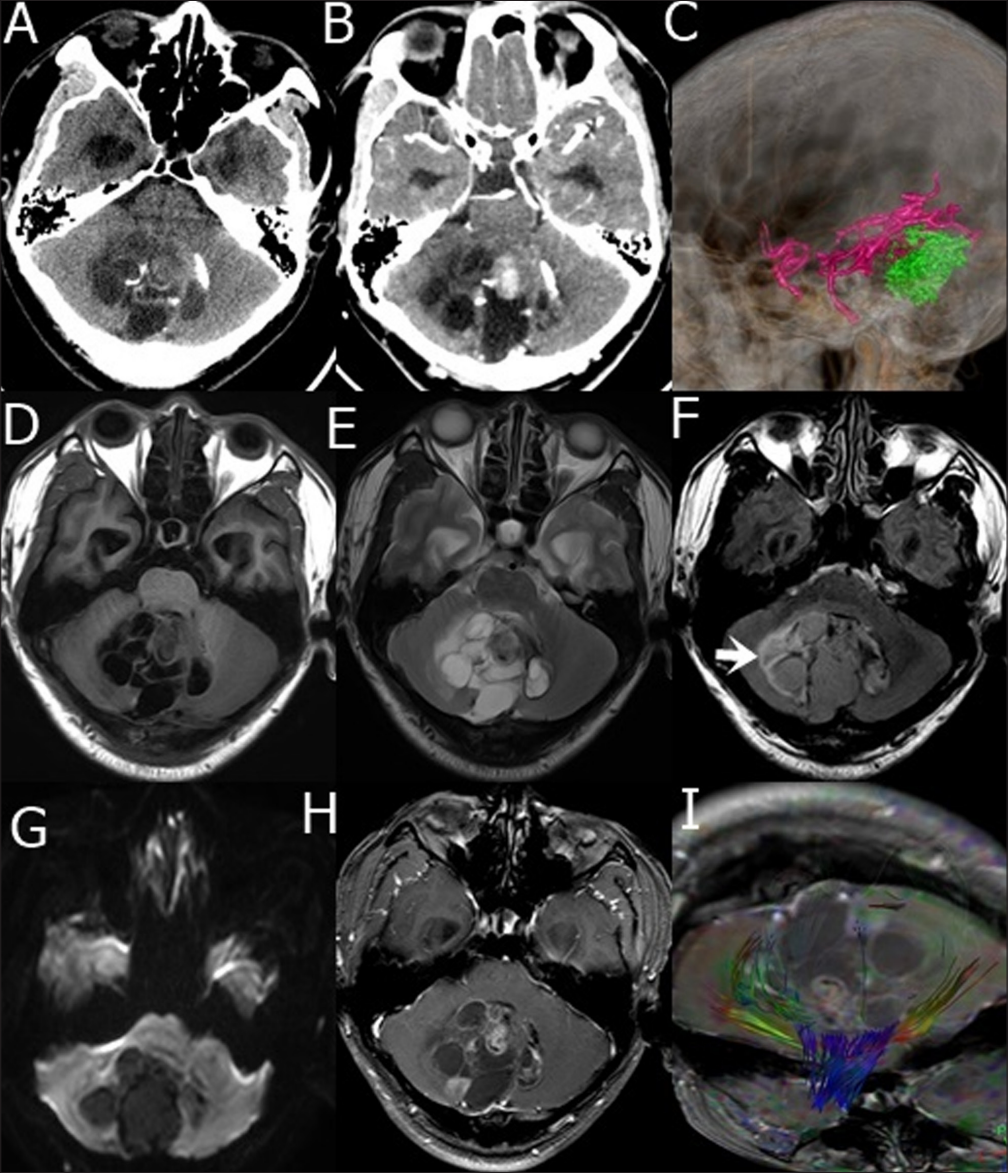
**A–I, male, 29 years old, DNET was present in the cerebellar vermis. A.** CT plain scan of cerebellar vermis showed low, slightly high mixed density, and scattered calcification; **B.** enhanced CT scan showed low-density areas without enhancement, solid nodules, septa, and cyst wall showed uneven enhancement; **C.** CTA branching arteries were seen at edge of tumors; **D.** T1WI tumor shows low and iso- mixed signal on T1WI; **E.** T2WI tumor shows high and iso- signal on T2WI; **F.** FLAIR tumor shows slightly hyperintensity, surrounded by high signal (arrow); **G.** DWI tumor shows low signal; **H.** Enhanced MRI the solid nodules, septa, and cyst walls were heterogeneously enhanced; **I.** DTI white matter fiber bundles were compressed and displaced.

**Figure 2:**
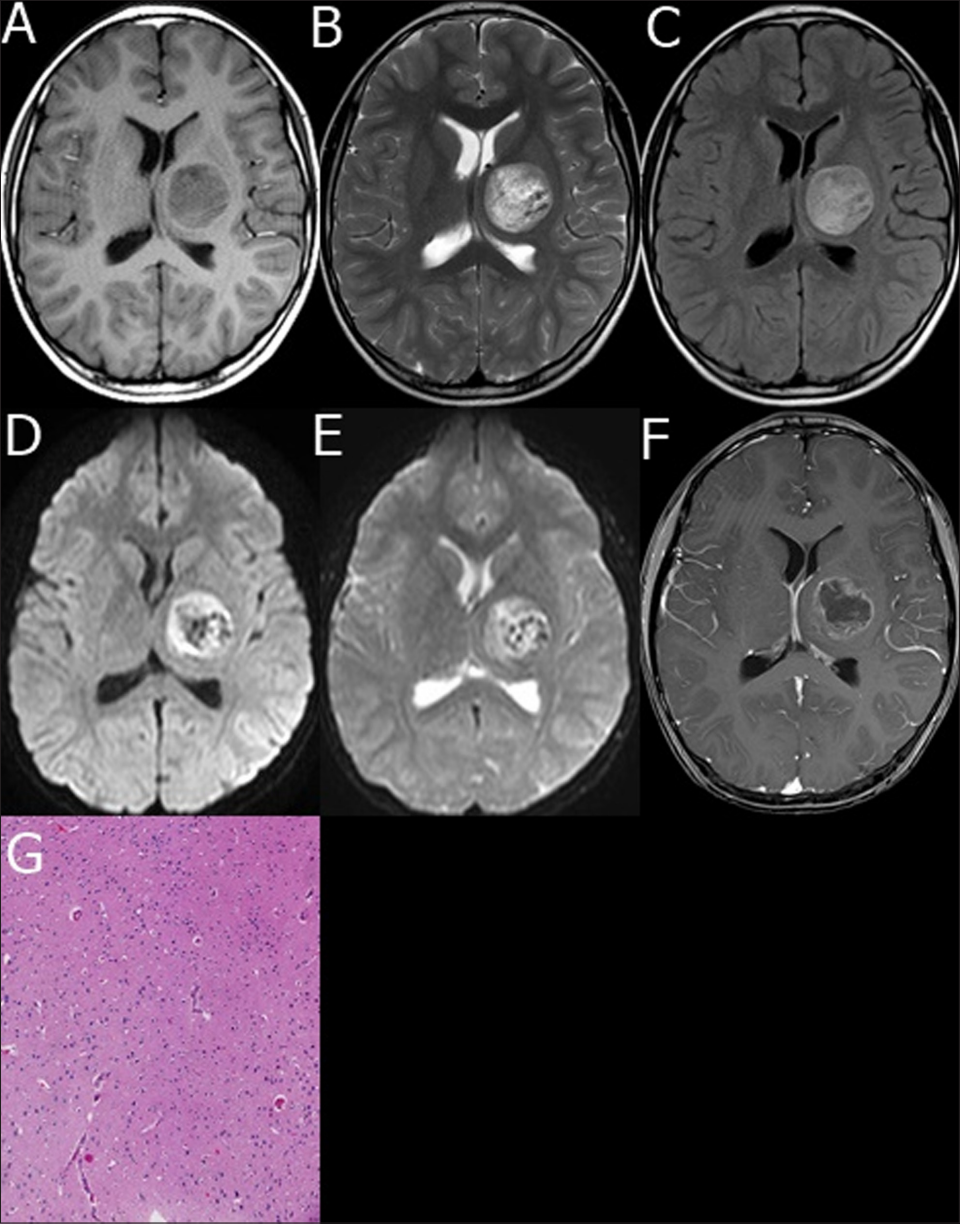
**A–G male, 8 years old, with DNET in the left basal ganglia. A.** T1WI left basal ganglia tumor showed iso- and low mixed signal; **B.** T2WI tumor showed iso- and slightly high and low mixed signal; **C.** FLAIR tumor showed iso- and slightly high mixed signal; **D.** DWI tumor showed iso- and low mixed signal; **E.** ADC diagram tumor showed high signal intensity; **F.** enhanced MRI of tumor showed annular enhancement; **G.** pathological images showed that the tumor tissue was formed by abundant mucous matrix and microcapsules. The morphology was similar to astrocytes and oligodendrocytes, and a few neurons were seen with slightly increased number of glial cells (HE, ×200).

The lesions in all six DNET patients were solitary, and lesions in one case was located in the right parietal lobe, one case in the right frontal lobe, one case in the cerebellar vermis (Figure 1A–I), one case in the right temporo-parietal occipital lobe, one case in the left basal ganglia (Figure 2A–G), and one case in the pineal gland. The diameters of lesions were range from 2.3 to 8.1 cm. All lesions in the six cases were cystic solid with a predominantly cystic component. The thickness of the cyst wall was about 0.2–0.4 cm. Multiple irregular separations within the sac were observed, and the thickness of the separation was about 0.2 to 0.5 cm. Single or multiple irregular soft tissue nodules with diameter of 0.5 to 2.6 cm were seen growing into the cystic cavity at the walls of the sac, with a clear boundary and no surrounding edema (Figures 1A~I, 2A~F). Nodular and short strip calcifications were scattered in the cyst wall or solid component in 4 cases (Figures 1A, 1B, 1D–F).

### 2.2 CT manifestations

The cystic components of the lesions on plain CT scan showed low density, the solid nodules, septa, and cyst walls were slightly high-density, and the calcification appeared high-density (Figure 1A). Enhanced CT scanning showed that cystic components was not strengthened, and solid nodules, divisions and sac walls were heterogeneously strengthened (Figure 1B). CTA showed multiple branching arteries (Figure 1C).

### 2.3 MRI manifestations

The cystic components of the lesions on plain MRI scanning showed low signal on T1WI and high signal on T2WI. Solid nodules, septa, and cyst walls of the lesions on T1WI and T2WI sequences showed equal or slightly high signal, and calcifications showed low signal (Figures 1D–F, 2A–C); FLAIR sequence showed slightly hyperintensity (Figures 1F, 2C); DWI lesions showed low signal in cystic components, solid nodules, septa, and cyst walls showed iso or slightly hyperintensity (Figures 1F, 2C), apparent diffusion coefficient (ADC) images showed high-intensity signal (Figure 2E); Diffusion tensor imaging (DTI) white matter fiber bundles were compressed and displaced, partially interrupted and reduced (Figure 1I); the cystic components were not enhanced on enhanced MRI scan, and the solid nodules, septa, and cyst walls showed heterogeneous enhancement (Figures 1H, 2F).

### 2.4 Surgery and pathology

During the operation, intracranial gray-yellow nodule-like tissue was seen occupying space. Some pale-yellow liquid was seen in the outflow. Three cases had no capsule, one case had pseudocapsule, and two cases had formed capsule, which was clearly demarcated with the surrounding brain tissue and with blood supply. Specimens from all cases were fixed with 4% formaldehyde, dehydrated in graded ethanol, embedded in paraffin, sectioned at 4 mm, and were stained with hematoxylin and eosin or stained immunohistochemically followed by observation under light microscope. Tumor tissue is formed by mucus-enriched matrix and microcystic cavity, with similar morphology to astrocytes and oligodendrocytes (Figure 2F). Immunohistochemistry in all lesions with antibodies as follows: GFAP (diffuse+), S-100 (diffuse+), SYN (+), NSE (+), CD34 (+), NF (+).

## 3 Discussion

### 3.1 Clinical manifestations and pathology

DNET is a rare tumor of the central nervous system, disease onset is before the age of 20 years in 90% of patients, and it is more common in children and adolescents, slightly more in men than in women. The average age of onset is 14.6 years in adolescents and 8.1 years in children [2]. There are four males and two females in our study. The oldest was 29 years old and the youngest was 5, with average age of 14.2 years old, consistent with the reports in the literature. DNETs are more common in the supratentorial cortex, the temporal lobe is the most common part of occurrence, followed by the frontal lobe, parietal lobe and occipital lobe. It can also occur in other parts, such as the basal ganglia, cerebellum, caudate nucleus and brain stem. Intractable epilepsy is almost the only symptom of DNET [5]. DNET has been proved to be the second most common epileptogenic tumor in children, following ganglion cell glioma [4]. In our study, lesions in one case was located in the right parietal lobe, one case in the right frontal lobe, one case in the cerebellar vermis, one case in the right temporo-parietal occipital lobe, one case in the left basal ganglia (Figure 2A–E), and one case in the pineal gland. Five patients visited hospital with drug refractory epilepsy, and one case visited with dizziness, headache and vomiting, consistent with the previous report.

DNET tissue originates from the second germinal layer during the development of the central nervous system that includes outer cerebellar granules, subpial granules, hippocampal dentate gyrus and subependymal layer, and pial granules are the most likely source. The typical pathological feature of DNET is specific glial neuron components (SGE) which is composed of different proportions of oligodendrocytes, astrocytes and neurons, and there is a rich mucus matrix between the cells. Morphologically, DNET can be divided into mononodular, multinodular, and diffuse types. According to the tissue alternations, it is divided into simple type, complex type and non-specific type: (1) simple type: the tumor is only composed of specific glial neuron components; (2) complex type: the tumor is composed of specific glial neuron components, neuroglial nodules and focal dysplastic cerebral cortical components; (3) non-specific: lack of typical specific glial neuron components [6]. In addition, some scholars have clearly defined DNET located in the septum pellucidum as a distinct type of lesion and new entity the so called myxoid glioneuronal tumors [7].

### 3.2 Imaging features

CT manifestations: DNETs are usually located in the supratentorial cerebral cortex or subcortex, with clear demarcation and low-density cystic lesions. When solid nodules or septations are obvious, they can appear as low-density, iso-density/slightly high-density mixed cystic and solid lesions. Patchy and nodular calcifications were seen in 20% of the lesions, bleeding was rare, and there was no edema around the tumor. Enhanced scan showed uneven enhancement in solid nodules, septa or the cyst wall, but no enhancement of the cystic components. All the cases in our group showed mixed cystic and solid lesions with low density, iso-/slightly high density, and three cases were located in the supratentorial. Patchy and nodular calcifications were found in the lesions in three cases, which was basically consistent with the literature.

MRI manifestations: DNET is typically characterized by multiple cystic structures or multiple cystic degeneration which show low signal on T1WI and high signal on T2WI. They are mainly located in the cortex with clear boundaries and no edema around [8]. No obvious mass effect is associated with DNET. Enhanced scanning shows no enhancement in the cystic components, and the solid nodules and the separation are unevenly enhanced. It has been reported that the enhancement of the solid nodules in the lesions is caused by the destruction of the blood-brain screen due to the vascular arch structure in the tumor tissue or the repeated seizures of epilepsy, while the enhancement of the intratumoral septum is due to the existence of thin-walled branch vessels [9]; the enhancement of solid nodules is related to glial cells with angiogenesisthe and the enhancement of the intratumoral septum is due to the secondary inflammatory changes caused by pressure on the residual brain tissue [10]. Fernandez et al. [11] reported that the triangular shape of the tumor and the presence of intratumoral septum strongly suggest the diagnosis of DNET. The ‘triangle sign’ is a triangular shape of the tumor when it is located on the surface of the brain with the broad base facing outward and the tip facing inward, and it may be related to the radial distribution of the glial fibers. DNET showed slightly high signal in FLAIR sequence, and the edge of the lesion showed linear and ring-shaped high signal, with no enhancement or mild enhancement on enhanced scan, that is, ‘ring sign’. ‘Ring sign’ is a characteristic imaging manifestation of DNET, which may be related to the loose tissue surrounding the glial-neuronal component at the tumor edge [12]. All tumors in our group were cystic and solid masses, mainly with multiple cystic structures. Enhanced scanning showed solid nodules and septation enhancement. Two cases showed ‘ring sign’, consistent with previous. No ‘triangle sign’ were found, which may be related to the small number of cases.

DWI shows the Brownian motion of water molecules. Since different brain tumors have different cell densities and diffusion speeds of water molecules, DWI can reflect the density of tumor cells through the diffusion speed. When the tumor cell density is high and the extracellular space is small, the DWI shows high signal, and the ADC (apparent diffusion coefficient) value shows low signal. In our group, three cases showed low signal on DWI and high signal on ADC diagram. Two DWI showed mainly low signal with solid nodule and surrounding showing slightly high signal, whereas ADC showed slightly high signal and low signal were found in solid nodule and surrounding tissues.

DTI reflects the predominant direction of diffusion of water molecules in white matter and shows the course of white matter fiber tracts. It allows the observation of the spatial directionality and integrity of white matter fiber tracts, namely white matter tract imaging. White matter fiber tracts in all the cases of our study were reconstructed with three-dimensional images. The tracts around the lesions were mainly compressed and displaced, reflecting the benign characteristics of DNET.

### 3.3 Differential diagnosis

DNET needs to be differentiated from the following tumors: (1) Oligodendroglioma: the oligodendroglioma tends to occur in adults. More than half of them is located in the frontal lobe, curved band-like calcification are its characteristic manifestations. (2) Low-grade astrocytoma: it mostly occurs in children and adults aged 20–40 years and is often located in the deep white matter. Characteristic features include cystic degeneration, calcification, rare hemorrhage and edema, irregular shape, large range, rare occurrence of triangle sign, intratumoral septum, high signal of FLAIR sequence with surrounding ring. (3) Ganglion cell glioma: tumors are more common in adolescents, and usually located in the temporal lobe. The typical manifestation is a single large cyst with calcification of wall nodule. In addition, the MR spectroscopy manifestation of reduced N-acetyl aspartate peak and lack of choline peak can help to distinguish DNETS from other tumors in the brain [13].

### 3.4 Limitation

There were some limitations in this study. First, the number of cases was small, and statistics analysis were not made. The summary of DNET imaging characteristics was not comprehensive enough. Because the incidence rate of DNET is extremely low, it needs to continue to collect in the future research, so as to facilitate a more comprehensive summary. Second, the treatment of DNET was not discussed. The focus of this study is to discuss the imaging manifestations, so it is not discussed in depth.

In summary, DNET is a rare benign tumor of the central nervous system, DNET display characteristic imaging and histopathological manifestations. Correct diagnosis of DNET can reduce unnecessary radiotherapy and chemotherapy, and improve the quality of life of patients. In future work, we will continue to collect DNET cases, and compare imaging such as CT and MRI with histopathology, including sensitivity and specificity, positive and negative predictive values, in order to improve the diagnostic coincidence rate of DNET imaging. At the same time, DNET has the possibility of malignant transformation. Although this risk is very small, it can’t be predicted by using any available biomarkers at present. Therefore, early diagnosis and treatment of DNET can improve the quality of life of patients.
